# Predicting persistence of hallucinations from childhood to adolescence

**DOI:** 10.1192/bjp.2021.115

**Published:** 2021-12

**Authors:** Lisa R. Steenkamp, Henning Tiemeier, Laura M. E. Blanken, Manon H. J. Hillegers, Steven A. Kushner, Koen Bolhuis

**Affiliations:** Department of Child and Adolescent Psychiatry/Psychology, Erasmus MC Sophia Children's Hospital, the Netherlands; Department of Child and Adolescent Psychiatry/Psychology, Erasmus MC Sophia Children's Hospital, the Netherlands and Department of Social and Behavioral Sciences, Harvard T.H. Chan School of Public Health, USA; Department of Psychiatry, Erasmus University Medical Centre, the Netherlands

**Keywords:** Psychotic-like experiences, psychotic disorders, schizophrenia, epidemiology, risk assessment

## Abstract

**Background:**

Psychotic experiences predict adverse health outcomes, particularly if they are persistent. However, it is unclear what distinguishes persistent from transient psychotic experiences.

**Aims:**

In a large population-based cohort, we aimed to (a) describe the course of hallucinatory experiences from childhood to adolescence, (b) compare characteristics of youth with persistent and remittent hallucinatory experiences, and (c) examine prediction models for persistence.

**Method:**

Youth were assessed longitudinally for hallucinatory experiences at mean ages of 10 and 14 years (*n =* 3473). Multi-informant-rated mental health problems, stressful life events, self-esteem, non-verbal IQ and parental psychopathology were examined in relation to absent, persistent, remittent and incident hallucinatory experiences. We evaluated two prediction models for persistence with logistic regression and assessed discrimination using the area under the curve (AUC).

**Results:**

The persistence rate of hallucinatory experiences was 20.5%. Adolescents with persistent hallucinatory experiences had higher baseline levels of hallucinatory experiences, emotional and behavioural problems, as well as lower self-esteem and non-verbal IQ scores than youth with remittent hallucinatory experiences. Although the prediction model for persistence versus absence of hallucinatory experiences demonstrated excellent discriminatory power (AUC_-corrected_ = 0.80), the prediction model for persistence versus remittance demonstrated poor accuracy (AUC_-corrected_ = 0.61).

**Conclusions:**

This study provides support for the dynamic expression of childhood hallucinatory experiences and suggests increased neurodevelopmental vulnerability in youth with persistent hallucinatory experiences. Despite the inclusion of a wide array of psychosocial parameters, a prediction model discriminated poorly between youth with persistent versus remittent hallucinatory experiences, confirming that persistent hallucinatory experiences are a complex multifactorial trait.

## Background

Psychotic experiences, such as hallucinations, commonly occur in the general population.^1,2^ Their expression peaks in childhood with a prevalence of 17% and declines in adolescence to a prevalence of 7.5%,^3^ which suggests that childhood psychotic experiences are often transient phenomena. Importantly, persistent psychotic experiences are in particular associated with adverse outcomes, including psychotic and non-psychotic mental disorders, substance use and suicidality,^4–6^ and thus likely to be more clinically relevant than transient psychotic experiences. Timely and accurate identification of youth who will develop persistent psychotic experiences is therefore of great clinical importance.

To this end, it is necessary to identify clinical determinants of symptom persistence versus discontinuation. However, a recent review stated that, although many studies have compared youth with persistent psychotic experiences to those without psychotic experiences, there is a paucity of information regarding differences between youth with persistent versus remittent patterns.^7^ Previous attempts to discover robust predictors of persistent versus remittent psychotic experiences have thus far been unsuccessful, suggesting that additional efforts are required in prospective cohorts involving detailed phenotypic data of young people with psychotic experiences. In addition, with the growing pressure on child and adolescent mental health services, predictive models to assess individual risk for persistence of psychotic experiences may improve outcomes from a public mental health perspective.

## Aims

Using data from a prospective population-based cohort, we first aimed to describe the developmental course of hallucinatory experiences between the ages of 10 and 14 years by calculating persistence and incidence rates. Second, we sought to examine differences in psychosocial and neurodevelopmental characteristics, as well as a family history of psychopathology between youth with persistent versus remittent hallucinatory experiences. Third, we used a predictive modelling strategy to examine whether we could predict persistence versus remittance and persistence versus absence of hallucinatory experiences at an individual level.

## Method

### Study population

The present study was part of the Generation R Study, a population-based prospective cohort in Rotterdam, the Netherlands, in which pregnant women were included between 2002 and 2006.^8^ The authors assert that all procedures contributing to this work comply with the ethical standards of the relevant national and institutional committees on human experimentation and with the Helsinki Declaration of 1975, as revised in 2008. All study procedures were approved by the Medical Ethics Committee of the Erasmus University Medical Centre Rotterdam. Written informed consent was obtained from all children and parents.

### Attrition analyses

Baseline data on hallucinatory experiences were available for 4340 children, of whom *n* = 3473 had follow-up data on hallucinatory experiences. Adolescents who were lost to follow-up (*n* *=* 867) reported slightly lower rates of hallucinations at age 10 years (28.3% *v.* 32.0%; *χ^2^* *=* 4.26, *P* = 0.039), more often had parents of Non-Western national origin (*χ^2^* *=* 95.99, *P* < 0.001) and had mothers with lower educational attainment (*χ^2^* *=* 92.59, *P* < 0.001) than adolescents who were retained at follow-up.

### Measures of hallucinatory experiences – age 10 and 14 years

**Fig. 1:**
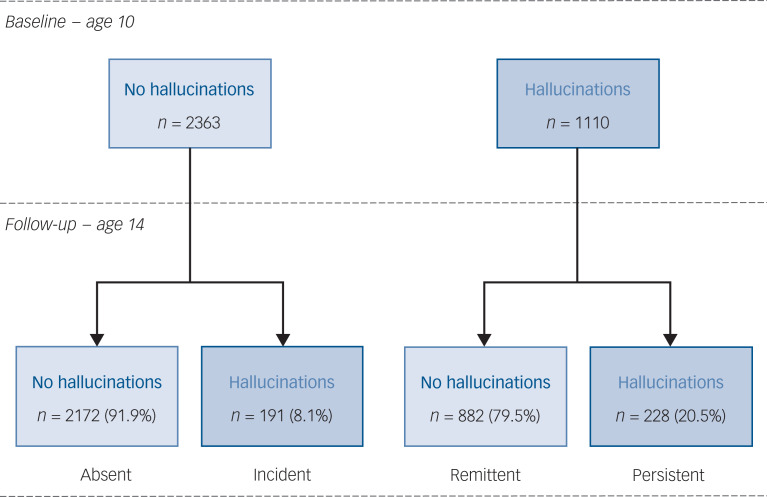
The course of hallucinatory experiences between ages 10 and 14 years (*n* *=* 3473).

Occurrence of hallucinatory experiences in the previous 6 months was assessed using two items from the Youth Self-Report (YSR): ‘I hear sounds or voices that other people think aren't there’ and ‘I see things that other people think aren't there’.^9–11^ Items were rated on a three-point scale: not at all (0), a bit (1), or clearly (2). Based on the sum scores, youth were classified into the following groups (Fig. 1).

- No hallucinatory experiences (a score of 0 on both time points): *n* *=* 2172 (63%)
- Persistent hallucinatory experiences (a score of ≥1 at baseline and ≥1 at follow-up): *n* *=* 228 (7%)
- Remittent hallucinatory experiences (a score of ≥1 at baseline and 0 at follow-up): *n* *=* 882 (25%)
- Incident hallucinatory experiences (a score of 0 at baseline and ≥1 at follow-up): *n* *=* 191 (5%)

### Measures of sociodemographic characteristics

Parental national origin was defined as Dutch if both parents were born in the Netherlands and as non-Dutch if at least one parent was born outside of the Netherlands. Children with parents of non-Dutch origin were further categorised into parental Other Western and parental Non-Western national origin. Maternal education level comprised low (high school or lower), medium (lower vocational education) and high (higher vocational education or university) education.

### Measures of child characteristics and parental psychopathology

The choice of predictors included was based on prior literature of risk factors for psychotic experiences and their persistence.^1,4,7,11–14^

#### Emotional and behavioural problems

At mean age 10 years, mothers completed the Child Behaviour Checklist (CBCL/6–18).^15^ We included the two broadband scales internalising and externalising problems, and the remaining subscales attention, thought and social problems. Although there is not a formal CBCL subscale for assessing sleep problems, we included a five-item sleep problems subscale in line with prior research.^16^ To assess self-reported internalising, externalising and attention problems, children completed the Brief Problem Monitor,^17^ which is an abbreviated version of the YSR.^15^ Self-reported sleep problems were derived from the Sleep Disturbance Scale for Children^18^ and adapted for the use in younger children.^16^

#### Childhood adversity

At mean age 10 years, mothers completed a face-to-face interview about 24 childhood adversities, such as physical or sexual maltreatment, parental divorce/separation, moving and neighbourhood problems.^19^ The perceived severity of each event was classified into none, a little, moderate or a lot. We summed all adversities with a moderate impact or higher, and created the following categories: no adversities, one or two adversities, and more than two adversities.

#### Self-esteem

At mean age 10 years, children completed the Dutch version of the Harter's Self-Perception Profile for Children,^20^ consisting of the subscales school competence, social acceptance, athletic competence and physical appearance. Subscale scores were combined into a total score.

#### Non-verbal IQ

At mean age 6 years, children were invited to the research centre and completed two subtests of the validated Dutch non-verbal intelligence test ‘the Snijders-Oomen Niet-verbale intelligentie Test-Revisie’.^21^ Scores were converted into a non-verbal IQ score based on age-specific reference scores from a Dutch population norm.^21^

#### Maternal history of psychiatric disorders

Maternal lifetime history of psychosis, depression and anxiety disorder was assessed using a self-report questionnaire during pregnancy. Paternal history of mental disorder was not included because of a high percentage of missingness.

#### Maternal and paternal psychopathology scores

Continuous maternal and paternal psychopathology scores were assessed at a mean child age of 10 years using the sum score of the subscales interpersonal sensitivity, anxiety, depression and hostility from the Brief Symptom Inventory.^22^

### Statistical analyses

Statistical analyses were performed using R version 4.1.0. First, we calculated persistence, remittance and incidence rates of hallucinatory experiences from age 10 to 14 years. Second, we evaluated main differences in child and parental characteristics between the four groups of hallucinatory experiences using χ^2^, Kruskal–Wallis or ANOVA tests. To assess pairwise comparisons, we followed up significant main differences using χ^2^-tests, *t*-tests or Mann–Whitney *U*-tests. In addition, we conducted multinomial logistic regression analyses to assess the associations of child and parental characteristics with the four different trajectories of hallucinatory experiences. To assess associations between characteristics and group membership of the persistent versus remittent group, we additionally conducted binary logistic regression analyses. Psychopathology scores were standardised and square root transformed to reduce positive skewness. We used a false discovery rate correction to control for type I errors.

Third, we evaluated a prediction model for persistence versus remittance using multivariable logistic regression. This analysis included all children who endorsed hallucinatory experiences at age 10 years (*n* = 1110). The outcome of interest was persistent (*n* *=* 228) versus remittent (*n* *=* 882) hallucinatory experiences at age 14 years. Discrimination was assessed using the *c*-statistic, which is identical to the area under the curve (AUC) for binary outcomes. Sensitivity and specificity were calculated based on the optimal cut-off point defined by the Youden index.^23^ Furthermore, we examined Nagelkerke's *R*^2^ to assess the proportion of explained variation. The model was internally validated using bootstrapping (500 replications), which resulted in an optimism-corrected AUC.^24^ Missing data on predictors were handled with multiple imputations using MICE. In a sensitivity step, we repeated the analysis using a stricter cut-off for hallucinatory experiences (score of ≥2, *n* *=* 513).

Finally, we conducted a sensitivity analysis using absent (*n* *=* 2172) rather than remittent hallucinatory experiences as the reference outcome for persistent hallucinatory experiences. In contrast to the first prediction model, this model is less likely to be applied in clinical settings, but provides an indication of the relevance, validity and appropriateness of the psychosocial parameters for predicting hallucinatory experiences more generally.

## Results

### Study population characteristics

Table 1 presents the descriptive characteristics of adolescents with different longitudinal patterns of psychotic experiences (*n* = 3473; see Supplementary Table 1 available at https://doi.org/10.1192/bjp.2021.115 for descriptive characteristics of the overall study population). The mean follow-up interval was 3.8 years. Between the ages 10 and 14 years, the prevalence of auditory and visual hallucinations dropped significantly, from 26.7% to 9.4% and from 17.0% to 6.9%, respectively.

**Table 1:** Sociodemographic, child and parental characteristics of children with longitudinal patterns of hallucinatory experiences (*n* = 3473)

	*n*	Pattern of hallucinatory experiences			
		Absent (*n* = 2172)	Persistent (*n* = 228)	Remittent (*n* = 882)	Incident (*n* = 191)
*Sociodemographic characteristics*					
Gender, % female	3473	51.5^a^	50.9^a^	50.7^a^	55.0^a^
Parental national origin, %	3456				
Dutch		70.7^a^	66.5^a,b^	71.1^a^	58.7^b^
Other Western		8.1	11.5	9.4	15.4
Non-Western		21.2	22.0	19.5	25.9
Maternal education, %	3304				
Low		12.5^a^	13.5^a^	11.6^a^	16.9^a^
Medium		27.4	32.1	26.5	28.6
High		60.1	54.4	61.9	54.5
*Child characteristics*					
Severity of hallucinations age 10 years, median (IQR)	3473	**–**	2.0 (1.0)^b^	1.0 (1.0)^a^	**–**
Multisensory hallucinations age 10 years, % yes	3473	**–**	48.2^b^	34.0^a^	**–**
Internalising problems age 10 years, median (IQR)					
Maternal report	3356	3.0 (5.0)^a^	5.0 (5.0)^c^	4.0 (6.0)^b^	3.0 (5.0)^b^
Self-report	3466	1.0 (3.0)^a^	3.0 (4.0)^d^	3.0 (3.0)^c^	2.0 (2.0)^b^
Externalising problems age 10 years, median (IQR)					
Maternal report	3355	2.0 (4.1)^a^	3.0 (6.0)^b^	3.0 (6.0)^b^	2.0 (5.2)^b^
Self-report	3458	1.0 (3.0)^a^	3.0 (3.0)^d^	2.0 (2.5)^b^	2.0 (3.0)^c^
Attention problems age 10 years, median (IQR)					
Maternal report	3355	2.0 (4.0)^a^	4.0 (4.0)^c^	3.0 (5.0)^b^	3.0 (4.0)^b,c^
Self-report	3467	2.0 (3.0)^a^	5.0 (3.3)^c^	4.0 (3.0)^b^	4.0 (4.0)^b^
Sleep problems age 10 years					
Maternal report, median (IQR)	3350	0.0 (1.0)^a^	1.0 (2.0)^c^	1.0 (2.0)^b^	0.0 (1.0)^b^
Self-report, mean (s.d.)	3415	10.4 (2.4)^a^	12.1 (2.5)^c^	11.6 (2.4)^b^	11.3 (2.4)^b^
Thought problems age 10 years, median (IQR)	3351	1.0 (2.0)^a^	1.0 (4.0)^c^	1.0 (3.0)^b^	1.0 (2.0)^b^
Social problems age 10 years, median (IQR)	3353	1.0 (2.0)^a^	1.0 (3.7)^c^	1.0 (3.0)^b^	1.0 (2.4)^b,c^
Self-esteem age 10 years, mean (s.d.)	3461	46.6 (3.9)^a^	43.4 (4.7)^c^	44.4 (4.4)^b^	44.9 (4.6)^b^
Childhood adversity, %	3364				
No adversities		74.9^a^	62.1^b^	65.4^b^	64.1^b^
1 or 2 adversities		21.0	28.6	28.6	26.1
>2 adversities		4.1	9.3	6.0	9.8
Non-verbal IQ age 6 years, mean (s.d.)	3043	104.5 (14.5)^a^	101.8 (13.4)^b^	104.6 (14.3)^a^	102.0 (14.1)^b^
*Parental psychopathology*					
Maternal history of mental disorders, % yes	2707	31.2^a^	40.1^b^	33.9^a,b^	35.6^a,b^
Maternal psychopathology (at child age 10 years), median (IQR)	3326	0.50 (0.95)^a^	0.62 (1.32)^b^	0.53 (1.05)^b^	0.62 (1.13)^a,b^
Paternal psychopathology (at child age 10 years), median (IQR)	2865	0.37 (0.78)^a^	0.37 (0.93)^b^	0.37 (0.73)^b^	0.33 (0.63)^a^

IQR, interquartile range. Statistically significant differences (*P* < 0.05) are shown by different superscript letters (for example four different superscript letters indicate that each of the groups are statistically different from each other: d > c > b > a). If two scores have the same superscript letter (for example ‘a’), these scores are not statistically different.

### Persistence, remittance and incidence rates

The persistence rate of hallucinatory experiences was 20.5% (Fig. 1). In other words, hallucinatory experiences were transient for the majority of 10-year-old children (remittance rate of 79.5%). The incidence rate of new-onset hallucinatory experiences was 8.1%.

### Persistent versus remittent hallucinatory experiences

#### Sociodemographic characteristics

We did not find differences in gender, maternal education level or parental national origin between youth with persistent versus remittent hallucinatory experiences (Tables 1 and 2).

**Table 2:** Associations of sociodemographic, child and parental characteristics with longitudinal patterns of hallucinatory experiences (*n* = 3473)

	Pattern of hallucinatory experiences				
	Absent (*n* = 2172)	Persistent (*n* = 228)	Remittent (*n* = 882)	Incident (*n* = 191)	Persistent versus remittent^a^
	OR (95% CI)	OR (95% CI)	OR (95% CI)	OR (95% CI)	OR (95% CI)
*Sociodemographic characteristics*					
Gender (female)	Reference	0.97 (0.74–1.28)	0.97 (0.83–1.13)	1.15 (0.85–1.55)	1.01 (0.75–1.35)
Parental national origin^b^					
Other Western	Reference	1.50 (0.96–2.35)	1.16 (0.88–1.53)	**2.28** (**1.47–3.54)**	1.29 (0.79–2.06)
Non-Western	Reference	1.11 (0.79–1.55)	0.91 (0.75–1.12)	**1.47** (**1.04–2.10)**	1.21 (0.84–1.73)
Maternal education^c^					
Medium	Reference	1.08 (0.69–1.71)	1.04 (0.79–1.38)	0.78 (0.48–1.25)	1.04 (0.64–1.72)
High	Reference	0.84 (0.55–1.29)	1.11 (0.86–1.43)	0.67 (0.44–1.03)	0.75 (0.48–1.21)
*Child characteristics at baseline (age 10),* *unless otherwise specified*					
Severity of hallucinations	Reference	**–**	**–**	**–**	**1.76** (**1.34–2.30)**
Multisensory hallucinations (yes)	Reference	**–**	**–**	**–**	**1.81** (**1.35–2.43)**
Internalising problems					
Maternal report	Reference	**1.69** (**1.47–1.94)**	**1.40** (**1.29–1.52)**	**1.30** (**1.12–1.52)**	**1.20** (**1.04–1.39)**
Self-report	Reference	**3.13** (**2.63–3.71)**	**2.09** (**1.91–2.28)**	**1.42** (**1.21–1.65)**	**1.48** (**1.24–1.76)**
Externalising problems					
Maternal report	Reference	**1.45** (**1.27–1.66)**	**1.31** (**1.21–1.42)**	**1.29** (**1.11–1.50)**	1.10 (0.96–1.27)
Self-report	Reference	**1.91** (**1.64–2.21)**	**1.47** (**1.36–1.60)**	**1.20** (**1.04–1.40)**	**1.28** (**1.10–1.50)**
Attention problems					
Maternal report	Reference	**1.77** (**1.53–2.05)**	**1.42** (**1.31–1.54)**	**1.44** (**1.24–1.69)**	**1.23** (**1.06–1.44)**
Self-report	Reference	**2.44** (**2.05–2.91)**	**1.93** (**1.76–2.12)**	**1.66** (**1.41–1.97)**	**1.25** (**1.05–1.51)**
Sleep problems					
Maternal report	Reference	**1.53** (**1.34–1.74)**	**1.29** (**1.19–1.39)**	**1.23** (**1.06–1.43)**	**1.18** (**1.03–1.36)**
Self-report	Reference	**2.01** (**1.73–2.34)**	**1.68** (**1.54–1.82)**	**1.48** (**1.27–1.73)**	**1.21** (**1.03–1.42)**
Thought problems	Reference	**1.58** (**1.38–1.81)**	**1.34** (**1.24–1.45)**	**1.34** (**1.15–1.55)**	1.17 (1.02–1.35)
Social problems	Reference	**1.54** (**1.34–1.76)**	**1.29** (**1.20–1.40)**	**1.31** (**1.12–1.52)**	**1.19** (**1.03–1.37)**
Self-esteem	Reference	**0.48** (**0.43–0.55)**	**0.58** (**0.53–0.63)**	**0.64** (**0.56–0.75)**	**0.82** (**0.72–0.94)**
Childhood adversity^d^					
1 or 2 adversities	Reference	**1.64** (**1.20–2.24)**	**1.56** (**1.30–1.88)**	1.45 (1.02–2.06)	1.05 (0.75–1.46)
>2 adversities	Reference	**2.72** (**1.64–4.52)**	**1.67** (**1.16–2.39)**	**2.79** (**1.62–4.79)**	1.63 (0.93–2.77)
Non-verbal IQ (age 6, per 10 points)	Reference	**0.88** (**0.79–0.97)**	1.00 (0.95–1.06)	0.89 (0.80–1.00)	**0.87** (**0.78–0.97)**
*Parental psychopathology*					
Maternal history of mental disorders (yes)	Reference	**1.48** (**1.09–2.01)**	1.13 (0.94–1.37)	1.22 (0.86–1.74)	1.31 (0.94–1.81)
Maternal psychopathology score at baseline	Reference	**1.29** (**1.13–1.47)**	**1.16** (**1.07–1.25)**	1.14 (0.98–1.33)	1.12 (0.97–1.29)
Paternal psychopathology score at baseline	Reference	**1.26** (**1.09–1.45)**	**1.12** (**1.03–1.22)**	0.97 (0.82**–**1.15)	1.13 (0.96–1.33)

False discovery rate-corrected significant associations (*P* *<* 0.05) are expressed in bold. Continuous predictors are standardised (mean 0, s.d. = 1), with the exception of IQ.

a. Binomial logistic regressions comparing the persistent and remittent (=reference) group.

b. ‘Dutch origin’ is reference group.

c. ‘Low education’ is reference group.

d. ‘No adversities’ is reference group.

#### Emotional and behavioural problems

Adolescents with persistent hallucinatory experiences had higher baseline levels of internalising, externalising, attention, social and sleep problems than adolescents with remittent hallucinatory experiences (Table 1). This finding was largely consistent across self-reports and mother reports (odds ratios (ORs) ranging from 1.10 to 1.48; Table 2). Furthermore, children with more severe hallucinatory experiences (OR = 1.76, 95% CI 1.34–2.30; Table 2) or multisensory (auditory and visual) rather than unisensory (auditory or visual) hallucinatory experiences (OR = 1.81, 95% CI 1.35–2.43; Table 2) at baseline had higher odds of developing persistent hallucinatory experiences.

#### Self-esteem

Children with a higher baseline level of self-esteem were more likely to remit than persist in their expression of hallucinatory experiences (OR = 0.82, 95% CI 0.72–0.94; Table 2).

#### Childhood adversity

Although the prevalence of childhood adversity was highest in the persistent group (37.9%; Table 1), the level of childhood adversity did not differ significantly between persistent and remittent patterns (Tables 1 and 2).

#### Non-verbal IQ

Youth with persistent hallucinatory experiences displayed an approximately 2.5 point lower non-verbal IQ score in early childhood than youth with remittent hallucinatory experiences (IQ = 101.8 *v*. 104.6; OR = 0.87, 95% CI 0.78–0.97; Tables 1 and 2).

#### Parental psychopathology

We did not find differences in maternal history of mental disorders, or maternal and paternal psychopathology scores between the persistent and remittent groups (Tables 1 and 2).

### Individual-level prediction of persistence

We examined whether we could predict which children would persistently endorse hallucinatory experiences at follow-up four years later (persistent: *n* *=* 228, remittent: *n* = 882). The explained variation of the prediction model was 7.4% and the AUC was 0.66 (*P* < 0.001), indicating poor discrimination between persistence and remittance (Supplementary Table 2). Sensitivity and specificity as calculated by the Youden index were 68.8% and 54.4%, respectively. Internal validation analysis resulted in an optimism-corrected AUC of 0.61.

In a sensitivity analysis, we assessed a prediction model using a stricter cut-off for hallucinatory experiences (persistent: *n* *=* 55, remittent: *n* = 458), which, similar to the previous model, resulted in poor discriminatory power (AUC-_corrected_ = 0.67).

**Fig. 2:**
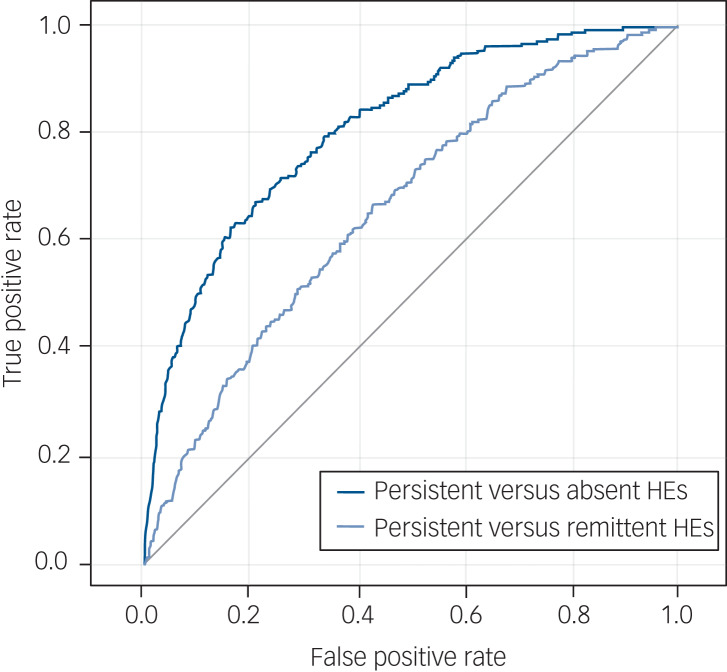
Plot of receiver operating characteristic curves of the two prediction models for persistent hallucinatory experiences (HEs): persistence versus remittance (area under the curve (AUC) = 0.66) and persistence versus absence (AUC = 0.81).

In another sensitivity analysis, we evaluated a prediction model that aimed to distinguish between youth with persistent versus absent – rather than remittent – hallucinatory experiences (persistent: *n* = 228, absent: *n* = 2172; Supplementary Table 3). Although this model does not directly answer our research question (i.e. estimating risk for persistence versus remittance), its predictive ability provides an indication of the validity of the included predictors. This model explained 23.8% of the variance and had excellent discriminatory power with an AUC of 0.81 (*P* < 0.001; sensitivity 84.4%, specificity 61.3%; AUC-_corrected_ = 0.80). The receiver operating characteristic curves of the persistent–remittent and persistent–absent prediction models are presented in Fig. 2.

## Discussion

In this prospective population-based study, we explored the developmental course of hallucinatory experiences between ages 10 and 14 years. Approximately 80% of youth with hallucinatory experiences at baseline remitted within the 4-year follow-up period. Adolescents with persistent hallucinatory experiences were characterised by a higher burden of baseline impairments across various domains, including more severe hallucinatory experiences and multi-informant-rated mental health problems, lower levels of self-esteem and lower non-verbal IQ scores. However, despite having a large sample size and using a wide variety of demographic and mental health parameters, the prediction model exhibited poor discrimination between persistent and remittent patterns of hallucinatory experiences. Although our findings were unsuccessful regarding individual-level prognosis of hallucinatory experience persistence versus remittance, several functional impairments distinguishing youth with persistent versus remittent hallucinatory experiences were clearly identifiable at a group level, which is relevant from public mental health and neurodevelopmental perspectives.

### Developmental course of hallucinatory experiences

Prior evidence suggests that the expression of psychotic experiences peaks in childhood and declines with advancing age.^3,12,14,25^ The findings of the present study strongly support this developmental trajectory by showing that the prevalence of hallucinatory experiences in childhood was approximately 2.5 times higher than in adolescence and approximately a fifth of children (20.5%) persisted in their endorsement of hallucinatory experiences from childhood into adolescence. Consequently, the majority of childhood hallucinatory experiences are transient phenomena, which may be clinically benign with spontaneous remission. Conversely, we found that 8.1% of youth who did not report hallucinatory experiences in childhood subsequently reported hallucinatory experiences in adolescence. This estimate is in accordance with the incidence rates reported in prior population-based studies.^13,25^ Although these children did not endorse any hallucinatory experiences at baseline, they were already characterised by higher baseline levels of mental health problems, experienced more adverse life events, had lower non-verbal IQ scores and more often had parents of non-Dutch origin than their unaffected peers. Prior work suggested that adolescent-onset hallucinations may reflect a more severe underlying psychopathology and index a greater risk for serious mental health problems than hallucinations occurring in childhood only.^13,25^ Taken together, the age at occurrence is an important factor to take into consideration when assessing hallucinations and their clinical relevance.

### Persistent versus remittent hallucinatory experiences

Children were more likely to develop persistent hallucinatory experiences if they had more severe hallucinatory experiences or reported multisensory (auditory *and* visual) rather than unisensory (auditory *or* visual) hallucinatory experiences at baseline. These findings are in line with prior evidence showing that a higher frequency, more associated distress and poorer reality testing are associated with persistence of auditory hallucinations.^25,26^ This suggests that the attributes of psychotic experiences may have important value in persistence–risk evaluations. Furthermore, youth with persistent hallucinatory experiences displayed higher baseline levels of internalising, externalising, sleep and social problems, and lower levels of self-esteem than youth with remittent hallucinatory experiences. Given that previous studies have suggested that persistence is related to a greater likelihood of subsequent emotional and behavioural problems,^13,25^ it is likely that psychotic experiences and other common mental health problems influence one another in a bi-directional manner. Accordingly, persistence of mental health problems, including but not limited to psychotic experiences, may ultimately culminate in severe mental health problems later in life.

Childhood adversity is one of the most replicated risk factors for psychotic experiences,^27^ and has been associated with persistence.^1,28^ Although the prevalence of adverse life events was highest in the persistent group, prevalence rates did not significantly differ between persistent and remittent patterns. A potential explanation is that childhood adversity is a non-specific risk factor and therefore might poorly discriminate within a group of children that have (a certain level of) symptoms. This is supported by our finding that adolescents with any of the different trajectories of hallucinatory experiences (i.e., persistent, remittent and incident) had higher rates of adverse life events than those without hallucinatory experiences. In addition, adverse life events were retrospectively measured and often occurred years before the assessment of hallucinatory experiences, which makes it less likely to detect an association with trajectories of hallucinatory experiences, as opposed to the association with baseline symptoms.

Importantly, we found lower non-verbal IQ scores at age 6 years and higher baseline levels of both self- and mother-reported attention problems among youth with persistent versus remittent patterns of hallucinatory experiences. These findings extend prior evidence of decreased working memory performance in relation to persistence.^29^ Interestingly, in a prior study we did not find evidence for a relationship between non-verbal IQ at age 6 years and hallucinatory experiences at age 10 years.^10^ Together with the finding that the incident group also exhibited lower non-verbal IQ scores in early childhood, this may suggest that adolescent hallucinatory experiences index a greater neurodevelopmental vulnerability than childhood hallucinatory experiences. Future work is needed to examine whether other neurodevelopmental problems, such as impaired social cognition, may be predictive of hallucination persistence. This is based on evidence suggesting that impaired theory of mind is associated with increased risk of future delusions among children with auditory hallucinations.^30^ A link with social cognition would also extend the current findings of social problems in relation to persistence of hallucinations.

### Proneness–persistence–impairment model of psychosis

Overall, the current findings provide support to the proneness–persistence–impairment model of psychosis.^14^ This model suggests that psychotic experiences are more likely to become persistent if neurodevelopmentally predisposed individuals are exposed to a higher degree of stressors, which, in turn, increases the probability for transition to psychosis and other clinical outcomes, such as mental health service use. It is likely that an accumulation of genetic, neurocognitive and environmental risk factors account for the incidence and particularly persistence of psychotic experiences – especially if these manifest during sensitive developmental windows in childhood/adolescence.^14^ Persistent psychotic experiences may therefore reflect an underlying neurodevelopmental vulnerability, which is phenotypically expressed through neurocognitive impairments, decreased social skills and increased psychopathological risk, as shown in the current study. Evidence from twin studies has indicated a genetic component in the stability of psychotic experiences over time,^31^ and it has been reported that cumulative exposure to environmental risk factors (such as trauma, cannabis use and urbanicity) affect the likelihood that psychotic experiences become persistent.^28^

Future waves of the Generation R Study will permit further longitudinal assessment of these youth into middle/late adolescence, enabling us to examine the impact of age-specific risk factors, such as substance use and risk-taking behaviour, which may interact with a pre-existing neurodevelopmental vulnerability. In addition, longitudinal assessments throughout child and adolescent development will allow further investigation of the relationship between persistence and age at onset of hallucinations, as well as the clinical significance and aetiology of childhood-onset versus adolescent-onset persistent hallucinations, which may have important implications for the notion of a neurodevelopmental vulnerability to hallucinations.

### Individual risk prediction of symptom persistence

In addition to descriptive analyses, we evaluated a prediction model leveraging all psychosocial characteristics in the data-set to assess whether we could identify which children with hallucinatory experiences at 10 years of age would persistently endorse hallucinatory experiences four years later. The results suggest that, given the included parameters and within this sample, the model was not able to accurately predict which children are at higher risk for persistence. This does not imply that the included predictors or their assessments are of poor validity as these are supported by the excellent discriminatory power of the prediction model using absent rather than remittent hallucinatory experiences as the reference outcome.

A likely explanation for the poor discrimination despite multiple group-level differences is the large variance of the predictor variables relative to their differences between persistent and remittent groups. Congruently, small effect sizes are observed in nearly all studies comparing the clinical characteristics of these subgroups.^7,12,13^ In other words, although we were able to detect differences at a group level, predicting an individual's risk of persistence proved to be more difficult because of the large overlap in likely predictor values. This suggests that persistent hallucinatory experiences are a complex and multifactorial phenotype. In addition, the dynamic expression of hallucinatory experiences over time (i.e. instability) may have contributed to the poor predictive accuracy.

Suggestions for improving future prediction models of persistence may include:

- a shorter follow-up period (for example 1–2 years) or repeated measurements over a longer period,
- more in-depth information on hallucinatory experiences at baseline (for example frequency, multimodality and associated distress),
- information on substance use, which was not possible in our study because of the young age of participants at baseline, and
- non-clinical predictors, such as genetic data, neuroimaging and biological markers.

Although we are not aware of any prediction models for persistence of childhood psychotic experiences, one previous study has evaluated a prediction model for persistence in middle adolescence.^4^ This model had a slightly higher discriminatory power (AUC of 0.74) than the current prediction model, which might be explained by the different age range of study participants (15–17 years), shorter follow-up period (2 years), potential overfitting because of the high number of predictors relative to the number of participants and the absence of internal validation.^24^ Given the lack of prognostic studies to date, future efforts are needed to assess the potential of prediction models for persistent psychotic experiences.

### Predictive psychiatry

In comparison to other fields of medicine, predictive modelling in psychiatry is at an early stage. The multifactorial aetiology of psychiatric phenotypes, ethical considerations (such as stigmatisation) and a lack of implementation research have posed challenges for the utilisation of prediction models in psychiatry.^32^ Despite these challenges, early identification through risk prediction can guide evidence-based decision-making in clinical practice, particularly in the context of preventive interventions. This may substantially improve clinical and functional outcomes and could potentially even prevent or delay the development of mental illness. To date, the majority of studies in the field of ‘precision psychiatry’ have focused on predicting psychosis in clinical high-risk (CHR) populations.^32^ CHR criteria include subthreshold psychotic symptoms, overall functional impairment and help-seeking behaviour,^33^ and thus is markedly different from the concept of psychotic experiences in the general population.

There are encouraging reports that targeted early interventions improve outcomes and delay the onset of psychosis in CHR individuals,^34^ although some scholars question the population benefits of these strategies because of the low prevalence of individuals at CHR in the general population (the ‘prevention paradox’).^35^ Evaluating risk assessments in population-based samples and settings is therefore an important contribution to existing research in risk-enriched samples. Ultimately, using risk assessments in population settings (such as primary care or schools) may offer new opportunities to prevent adverse psychiatric outcomes, such as enduring and distressing psychotic experiences.

### Strengths and limitations

Although the present study had several strengths, including its prospective design with repeated measurements of hallucinatory experiences and multi-informant assessments of a range of relevant psychosocial characteristics, several limitations should be noted.

First, since the self-report questionnaire on psychotic experiences was restricted to hallucinations, our findings may not extend to the full range of psychotic experiences including delusions. However, self-reported auditory and visual hallucinations have the highest predictive power for clinician-confirmed psychotic experiences.^36^

Second, our self-report questionnaire of hallucinations may have led some children to misinterpret the questions, potentially resulting in an overestimation of the prevalence and remittance rates of childhood hallucinations, which may partly explain the large decline in prevalence and the low stability of hallucinations between childhood and adolescence. Such misinterpretations may be caused by the restricted questionnaire on hallucinations, which did not include examples or assessment of the children's understanding of the questions. Ideally, studies may consider using instruments that are specifically tailored for children, which also involve strategies to lower barriers for self-disclosure of hallucinations.^2^ Nevertheless, a recent study reported that the positive predictive value of self-reported psychotic experiences benchmarked against clinical interviews did not differ between ages 6 to 10 years and 11 to 14 years,^37^ thereby providing support for the validity of a brief and general questionnaire for assessing hallucinations in younger children.

Third, it may have been preferable to use a data-driven approach to identify trajectories of hallucinatory experiences (such as growth mixture modelling), but this was not feasible given that hallucinatory experiences were assessed at only two time points in the setting of a limited questionnaire regarding hallucinatory experiences.

### Implications

In conclusion, this study provides evidence for the dynamic and mostly transitory developmental expression of childhood hallucinatory experiences. Children and their parents who are concerned should be informed about the high prevalence rates and frequent spontaneous remission of psychotic experiences occurring in childhood. This knowledge may contribute to reduce the stigma of hallucinations. However, psychotic experiences that are distressing, frequent and persistent over time are likely to be indicative of an underlying neurodevelopmental vulnerability, which might, in turn, result in serious mental conditions later in life. Together, these findings highlight the relevance of assessing psychotic experiences prospectively across development.

## Data Availability

The data that support the findings of this study can be obtained upon request. Requests should be directed to the management team of the Generation R Study (secretariaat.genr@erasmusmc.nl), which has a protocol of approving data requests. Because of restrictions based on privacy regulations and informed consent of participants, data cannot be made freely available in a public repository.

## Supplementary material

For supplementary material accompanying this paper visit http://dx.doi.org/10.1192/bjp.2021.115. click here to view supplementary material
