# Supplementary material for: Predicting persistence of hallucinations from childhood to adolescence
Source: Br J Psychiatry. 2021 Dec;219(6):670–7. doi: 10.1192/bjp.2021.115 (PMC8674728; doi:10.1192/bjp.2021.115)
Supplement: Supplementary file 1 [file S000712502100115Xsup001.docx]

**Supplementary Material**

**Table S1.** Characteristics of the study population.

|  | Study population (*N*=3473) | | |
| --- | --- | --- | --- |
|  | Total *n* | % missing |  |
| *Sociodemographic characteristics* |  |  |  |
| Sex, % girls | 3473 | 0.0 | 51.5 |
| Parental national origin, % | 3456 | 0.5 |  |
| Dutch | 2415 |  | 69.9 |
| Other Western | 313 |  | 9.0 |
| Non-Western | 728 |  | 21.1 |
| Age (years) at first hallucination assessment, mean (SD) | 3473 | 0.0 | 9.8 (0.3) |
| Age (years) at second hallucination assessment, mean (SD) | 3473 | 0.0 | 13.6 (0.4) |
| Maternal educational level, % | 3304 | 4.9 |  |
| Low (no education – high school) | 415 |  | 12.6 |
| Medium (lower vocational education) | 910 |  | 27.5 |
| High (higher vocational education and university) | 1979 |  | 59.9 |
| *Child characteristics* |  |  |  |
| Internalizing problems age 10, median (IQR) |  |  |  |
| Maternal report | 3356 | 3.4 | 3.0 (6.0) |
| Self-report | 3466 | 0.2 | 2.0 (3.0) |
| Externalizing problems age 10, median (IQR) |  |  |  |
| Maternal report | 3355 | 3.4 | 2.0 (5.0) |
| Self-report | 3458 | 0.4 | 2.0 (3.0) |
| Attention problems age 10, median (IQR) |  |  |  |
| Maternal report | 3355 | 3.4 | 2.0 (4.0) |
| Self-report | 3467 | 0.2 | 3.0 (4.0) |
| Sleep problems age 10 |  |  |  |
| Maternal report, median (IQR) | 3350 | 3.5 | 0.0 (1.0) |
| Self-report, mean (SD) | 3415 | 1.7 | 10.9 (2.5) |
| Thought problems age 10, median (IQR) | 3351 | 3.5 | 1.0 (2.0) |
| Social problems age 10, median (IQR) | 3353 | 3.5 | 1.0 (2.0) |
| Self-esteem age 10, mean (SD) | 3461 | 0.3 | 45.7 (4.2) |
| Childhood adversity, % | 3364 | 3.1 |  |
| No adversities | 2389 |  | 71.0 |
| 1 or 2 adversities | 799 |  | 23.8 |
| >2 adversities | 176 |  | 5.2 |
| Non-verbal IQ age 6, mean (SD) | 3043 | 12.4 | 104.0 (14.3) |
| *Parental psychopathology* |  |  |  |
| Maternal history of mental disorders, % yes | 2707 | 22.1 | 32.8 |
| Maternal psychopathology, median (IQR) | 3326 | 4.2 | 0.53 (1.03) |
| Paternal psychopathology, median (IQR) | 2865 | 17.5 | 0.37 (0.83) |

**Table S2.** Prediction of persistence versus remittance of hallucinatory experiences (AUC=0.66, AUC-corrected=0.61).

| Predictor variable | *β* | SE | Wald *X^2^* | OR | (95% CI) | *P-value* | |  |
| --- | --- | --- | --- | --- | --- | --- | --- | --- |
| Intercept | -1.518 | 0.273 | -5.57 | 0.22 | (0.13–0.37) | <0.001 | |  |
| *Sociodemographic characteristics* |  |  |  |  |  |  | |  |
| Sex, female | -0.051 | 0.161 | -0.32 | 0.95 | (0.69–1.30) | 0.749 | |  |
| Parental national origin^a^ |  |  |  |  |  |  |  | |
| Other Western | 0.256 | 0.257 | 1.00 | 1.29 | (0.78–2.14) | 0.320 | |  |
| Non Western | -0.003 | 0.206 | -0.01 | 1.00 | (0.67–1.49) | 0.989 | |  |
| Maternal education^b^ |  |  |  |  |  |  |  | |
| Medium | 0.211 | 0.276 | 0.77 | 1.24 | (0.72–2.12) | 0.443 | |  |
| High | -0.056 | 0.258 | -0.22 | 0.95 | (0.57–1.57) | 0.830 | |  |
| *Child characteristics at baseline (unless otherwise specified)* | | | |  |  |  | |  |
| Hallucination severity^c^ | 0.193 | 0.075 | 2.57 | 1.21 | (1.05–1.40) | 0.010 | |  |
| Internalizing problems |  |  |  |  |  |  |  | |
| Maternal report | -0.050 | 0.115 | -0.44 | 0.95 | (0.76–1.19) | 0.663 | |  |
| Self-report | 0.225 | 0.091 | 2.48 | 1.25 | (1.05–1.50) | 0.013 | |  |
| Externalizing problems |  |  |  |  |  |  |  | |
| Maternal report | -0.202 | 0.117 | -1.73 | 0.82 | (0.65–1.03) | 0.084 | |  |
| Self-report | 0.169 | 0.089 | 1.90 | 1.18 | (0.99–1.41) | 0.057 | |  |
| Attention problems |  |  |  |  |  |  |  | |
| Maternal report | 0.141 | 0.116 | 1.22 | 1.15 | (0.92–1.44) | 0.224 | |  |
| Self-report | -0.032 | 0.100 | -0.32 | 0.97 | (0.80–1.18) | 0.748 | |  |
| Sleep problems^d^ | 0.040 | 0.085 | 0.47 | 1.04 | (0.88–1.23) | 0.635 | |  |
| Thought problems | 0.072 | 0.106 | 0.68 | 1.07 | (0.87–1.32) | 0.498 | |  |
| Social problems | 0.033 | 0.116 | 0.28 | 1.03 | (0.82–1.30) | 0.780 | |  |
| Self-esteem | 0.000 | 0.089 | 0.00 | 1.00 | (0.84–1.19) | 0.998 | |  |
| Childhood adversity^e^ |  |  |  |  |  |  |  | |
| 1 or 2 adversities | -0.095 | 0.180 | -0.53 | 0.91 | (0.64–1.30) | 0.599 | |  |
| >2 adversities | 0.125 | 0.306 | 0.41 | 1.13 | (0.62–2.07) | 0.682 | |  |
| Non-verbal IQ (age 6) | -0.161 | 0.089 | -1.80 | 0.85 | (0.71–1.02) | 0.072 | |  |
| *Parental psychopathology* |  |  |  |  |  |  |  |  |
| Maternal history of mental disorders (yes) | 0.214 | 0.182 | 1.17 | 1.24 | (0.87–1.77) | 0.241 | |  |
| Maternal psychopathology score | -0.006 | 0.085 | -0.07 | 0.99 | (0.84–1.17) | 0.944 | |  |
| Paternal psychopathology score | 0.126 | 0.080 | 1.57 | 1.13 | (0.97–1.33) | 0.116 | |  |

^a^ “Dutch origin” is reference group. ^b^ “Low education” is reference group. ^c^ Multimodality not included because of overlap with severity scale. ^d^ Only self-report items are included because mother-report items are a part of other included CBCL subscales. ^e^ “No adversities” is reference group. All continuous predictors are standardized (mean=0, SD=1). Missing predictors are imputed using multiple imputation.

**Table S3.** Prediction of persistence versus absence of hallucinatory experiences (AUC=0.81, AUC-corrected=0.80).

| Predictor variable | *β* | SE | Wald *X^2^* | OR | (95% CI) | *P-value* | |  |  |
| --- | --- | --- | --- | --- | --- | --- | --- | --- | --- |
| Intercept | -3.282 | 1.319 | -2.49 | 0.04 | (0.00–0.50) | 0.013 | |  |  |
| *Sociodemographic characteristics* |  |  |  |  |  |  | |  |  |
| Sex (female) | -0.060 | 0.158 | -0.38 | 0.94 | (0.69–1.28) | 0.706 | |  |  |
| Parental national origin^a^ |  |  |  |  |  |  |  | | |
| Other Western | 0.606 | 0.252 | 2.41 | 1.83 | (1.12–3.00) | 0.016 | |  |  |
| Non Western | 0.094 | 0.202 | 0.47 | 1.10 | (0.74–1.63) | 0.641 | |  |  |
| Maternal education^b^ |  |  |  |  |  |  |  | | |
| Medium | 0.396 | 0.275 | 1.44 | 1.49 | (0.87–2.55) | 0.149 | |  |  |
| High | 0.096 | 0.264 | 0.36 | 1.10 | (0.66–1.85) | 0.717 | |  |  |
| *Child characteristics at baseline (unless otherwise specified)* | | | |  |  |  | | |  |
| Internalizing problems |  |  |  |  |  |  |  | | |
| Maternal report | 0.010 | 0.022 | 0.47 | 1.01 | (0.97–1.05) | 0.639 | |  |  |
| Self-report | 0.313 | 0.040 | 7.76 | 1.37 | (1.26–1.48) | <0.001 | |  |  |
| Externalizing problems |  |  |  |  |  |  |  | | |
| Maternal report | -0.031 | 0.023 | -1.32 | 0.97 | (0.93–1.02) | 0.188 | |  |  |
| Self-report | 0.090 | 0.046 | 1.94 | 1.09 | (1.00–1.20) | 0.052 | |  |  |
| Attention problems |  |  |  |  |  |  |  | | |
| Maternal report | 0.068 | 0.035 | 1.96 | 1.07 | (1.00–1.15) | 0.051 | |  |  |
| Self-report | 0.096 | 0.040 | 2.38 | 1.10 | (1.02–1.19) | 0.018 | |  |  |
| Sleep problems^c^ | 0.101 | 0.035 | 2.92 | 1.11 | (1.03–1.18) | 0.004 | |  |  |
| Thought problems | 0.040 | 0.046 | 0.88 | 1.04 | (0.95–1.14) | 0.381 | |  |  |
| Social problems | -0.060 | 0.049 | -1.23 | 0.94 | (0.86–1.04) | 0.217 | |  |  |
| Self-esteem | -0.034 | 0.021 | -1.64 | 0.97 | (0.93–1.01) | 0.102 | |  |  |
| Childhood adversity^d^ |  |  |  |  |  |  |  | | |
| 1 or 2 adversities | -0.083 | 0.186 | -0.45 | 0.92 | (0.64–1.33) | 0.656 | |  |  |
| >2 adversities | -0.024 | 0.333 | -0.07 | 0.98 | (0.51–1.88) | 0.943 | |  |  |
| Non-verbal IQ (age 6) | -0.004 | 0.006 | -0.63 | 1.00 | (0.98–1.01) | 0.531 | |  |  |
| *Parental psychopathology* |  |  |  |  |  |  |  | | |
| Maternal history of mental disorders (yes) | 0.104 | 0.187 | 0.56 | 1.11 | (0.77–1.60) | 0.578 | |  |  |
| Maternal psychopathology score | 0.052 | 0.068 | 0.77 | 1.05 | (0.92–1.20) | 0.442 | |  |  |
| Paternal psychopathology score | 0.069 | 0.080 | 0.85 | 1.07 | (0.91–1.25) | 0.393 | |  |  |

^a^ “Dutch origin” is reference group. ^b^ “Low education” is reference group. ^c^ Only self-report items are included because mother-report items are a part of other included CBCL subscales. ^d^ “No adversities” is reference group. All continuous predictors are standardized (mean=0, SD=1). Missing predictors are imputed using multiple imputation.
